# Willingness of adolescent girls and young women to participate in future clinical trials of long-acting PrEP implants for HIV prevention, Kampala Uganda

**DOI:** 10.1371/journal.pgph.0005028

**Published:** 2025-08-08

**Authors:** Jane Frances Lunkuse, Grace Godfrey Sseremba, Candice Chetty-Makkan, Elizabeth Wahome, Matt A. Price, Yunia Mayanja

**Affiliations:** 1 Medical Research Council/Uganda Virus Research Institute and London School of Hygiene and Tropical Medicine Uganda Research Unit, Entebbe, Uganda; 2 Faculty of Health Sciences, University of the Witwatersrand, Johannesburg, South Africa; 3 Radcliffe Department of Medicine, University of Oxford, Oxford, United Kingdom; 4 International AIDS Vaccine Initiative, New York, New York, United States of America; 5 Department of Epidemiology & Biostatistics, University of California San Francisco, San Francisco, California, United States of America; 6 London School of Hygiene & Tropical Medicine, London, United Kingdom; Worcester Polytechnic Institute, UNITED STATES OF AMERICA

## Abstract

Adolescent girls and young women (AGYW) continue to face a significant risk of HIV infection, particularly as numerous experimental prevention products are in development. This study assessed the willingness to participate (WTP) in future clinical trials of long-acting HIV pre-exposure prophylaxis (PrEP) implants among AGYW at high risk of HIV infection in Kampala, Uganda. From January to October 2019, we conducted a cross-sectional study among AGYW aged 14–24 years. Interviewers _collected data on socio-demographics, substance use, sexual behavioural risk, contraceptive use and laboratory diagnosis of sexually transmitted infections (STIs). Participants were asked about their WTP in future clinical trials of a long-acting PrEP (LAP) implant. Multivariable logistic regression models were fitted to determine participant characteristics associated with WTP in future clinical trials of a PrEP implant. We enrolled 285 participants, with a median age of 20 years. Among them, 57.2% were single, 54.7% had completed at least secondary education, 92.6% engaged in transactional sex, 36.5% had multiple new male partners, 25.3% tested positive for STIs (chlamydia or gonorrhoea), and 15.4% reported drug use in the past three months. Nearly half (45.6%) of the AGYW expressed willingness to participate in a future HIV prevention study involving the LAP implant. Willingness to participate in a future HIV prevention study involving the LAP implant was higher among those with multiple new male partners (adjusted odds ratio [aOR] 1.84, 95%CI 1.09-3.11, *P* = 0.022) and those using contraceptives (aOR 1.69, 95% CI 1.00-2.85, *P* = 0.047) but lower among those with higher income levels (aOR 0.46, 95%CI 0.25-0.84, *P* = 0.013). These findings suggest that AGYW with higher HIV risk and those with prior contraceptive experience could potentially participate in future clinical trials of the LAP implant.

## Introduction

Globally, approximately 39.9 million people were living with HIV at the end of 2023, and an estimated 4000 adolescent girls and young women (AGYW) aged 15–24 years were diagnosed with HIV every week [1]. In Eastern and Southern Africa, AGYW remain at substantial risk of acquiring HIV, with four out of five new infections occurring in this age group annually [2]. Despite the availability of prevention interventions, AGYW are three times as likely to be living with HIV when compared to their male counterparts [3]. Key structural and behavioural factors contributing to the increased HIV risk among AGYW include poverty, gender inequalities linked to power imbalance between men and women, and lack of autonomy over their bodies and sexual decision-making [4].

Effective prevention strategies are critical for reducing HIV acquisition among AGYW, particularly those living or working in high-risk contexts. Currently, several HIV prevention methods are available including behavioural interventions such as HIV testing and counselling, abstinence, being faithful to sexual partners and condoms. Despite condoms being available, their uptake has remained low among AGYW, mainly due to lack of condom negotiation skills [5,6] given that it is a male-controlled method. The HIV prevention toolkit has evolved over the past decade with new biomedical HIV prevention methods such as oral pre-exposure prophylaxis (PrEP) demonstrating high effectiveness with good adherence. However PrEP uptake remains suboptimal among AGYW in sub-Saharan Africa, largely due to stigma, pill burden, lack of awareness of PrEP access points and high mobility [7–13]. Long-acting HIV prevention methods such as the dapivirine vaginal ring and injectable cabotegravir (CAB-LA), have shown efficacy in clinical trials, are recommended by the World Health Organization and have been incorporated into the Uganda National HIV Prevention and Treatment guidelines [14–16]. Yet their scale-up is limited due to factors such as cost and CAB-LA’s prolonged pharmacokinetic tail [17]. More recently, long-acting lenacapavir, a twice yearly injectable has shown 100% efficacy in early studies, although results are still under investigation [18]. Other novel products under development, such as broadly neutralising antibodies and the subdermal PrEP implants are promising. These will have a longer duration of protection, hence the lowest likelihood of non-compliance. The subdermal tenofovir alafenamide and cabotegravir biodegradable PrEP implants that have shown good safety profiles in pre-clinical studies will likely offer protection for up to 12 months [19,20]. Like contraceptive implants, HIV PrEP implants are intended to be inserted within the body and would not require frequent clinic visits, potentially improving adherence. However, once developed, these would have to demonstrate good safety profiles and high efficacy in clinical trials before being rolled out for use among eligible populations including AGYW at risk for HIV acquisition. The long-acting PrEP implant could serve as a valuable alternative for at-risk populations, particularly for those who are mobile, due to its anticipated longer duration of protection compared to current injectable PrEP options like lenacapavir, which provides up to six months of coverage. The PrEP implant provides a continuous and reliable method of HIV prevention, bridging the gap until advancements in vaccine technology are achieved. Given its expected high effectiveness, convenience, long-lasting protection, and discreet design, findings show that a PrEP implant has the potential to address the challenges of other existing biomedical HIV prevention products [21]. It is important to understand the willingness to participate (WTP) in such trials to inform future trial design once these products become available. Therefore, this study aimed to determine the factors associated with WTP in future clinical trials of a long-acting PrEP implant for HIV prevention among AGYW aged 14–24 years in Kampala, Uganda.

## Materials and methods

### Study design and setting

We performed a cross-sectional analysis of baseline data from a two-year cohort study among AGYW recruited from commercial hotspots (i.e., where sex work, alcohol and illicit drug were common) between 14 January 2019 and 17 October 2019. The study was conducted at the Good Health for Women Project (GHWP) clinic, established in 2008 in a peri-urban community in southern Kampala [22]. The clinic offered free services including HIV prevention and care, general health care and reproductive health services to eligible high-risk women and their regular male partners.

### Study population, sampling, and eligibility

The overall aim of the cohort study, entitled “Interventions for HIV Prevention among Adolescent girls and young women (IPAD),” was to assess knowledge and preferences for biomedical HIV prevention interventions and uptake of oral PrEP among AGYW at high risk of HIV infection in Kampala, Uganda. AGYW were enrolled in the cohort if they were HIV negative, sexually active in the past 3 months, willing to undergo study procedures, Hepatitis B immune or willing to receive Hepatitis B vaccination if not immune. Participants were excluded if pregnant or planning pregnancy in the next 12 months; had known allergy to components of oral PrEP drugs, contraceptives, or Hepatitis B vaccine; and reported or were diagnosed with an illness that would affect participation in the study. The study population and sampling for the cohort have previously been described [8].

### Participant education on future HIV prevention trials and WTP survey

Study staff reviewed hypothetical information on future HIV prevention trials of products, including a PrEP implant with study participants. The document included available information from pre-clinical studies [23] as follows: product form, mode of administration, duration of action, potential side effects and product demonstration using a contraceptive implant as a proxy. Clinical trial attributes were also described including randomisation, frequency of clinic visits, blood draws of 40–50 mls (8–10 teaspoons) for immune response assays, study visit duration of approximately 2 hours, requirements to use effective contraception and undergo regular HIV and pregnancy testing throughout the study period. Subsequently, staff administered a survey to evaluate participants’ hypothetical willingness to: participate in a clinical trial investigating a long-acting PrEP implant, attend study visits, accept randomization to either an active product or placebo, commit to the study for at least 3 years, use effective contraception, undergo regular pregnancy testing and if pregnant, accept withdrawal from the study to avoid harm. Study staff also provided participants with information on upcoming trials for future HIV vaccines and injectable PrEP options.

### Data collection

The WTP assessment took place at enrolment. Trained study staff collected data using standardized interviewer administered paper questionnaires.

Additional data collected included socio-demographic characteristics, sexual risk behaviour, contraceptive use, substance use and laboratory diagnosis of sexually transmitted infections (STIs). All data were double entered into an Open Clinica database by trained data entrants.

### Measurement of variables

#### Primary outcome.

The primary study outcome was the willingness of AGYWs to participate in future clinical trials of a long acting-PrEP (LAP) implant. This was measured as a binary outcome (yes, no). After receiving education on a hypothetical HIV prevention trial of a PrEP implant, participants were asked to respond to the following question:

*The implant would be inserted under your skin at study enrolment, would you be willing to be in a study for 3 years and visit the clinic regularly for assessments, for example every 3 months?*

Additional questions were also asked to explore the reasons for willingness or unwillingness to participate in a future implant trial.

#### Independent variables.

Socio-demographic variables included: age at enrolment in years, education level (none, primary, secondary and tertiary), marital status (married, separated/divorced, single/never married), main job (sex work, hospitality/entertainment, no job, other), number of biological children and weekly income.

Substance use: alcohol use in the past 12 months was assessed using the AUDIT tool (Alcohol Use Disorders Identification Test) [24] and scores were categorized as follows: 0, None; 1–7, low risk; 8–15, moderate risk/hazardous; ≥ 16, high risk/alcohol dependent. Drug use in the past one month (yes/no).

Sexual risk and reproductive health variables were assessed in the past 3 months and included: number of new sexual partners, condom use (yes/ no), contraceptive use (oral pills, injectable, implant, intra uterine device, condoms and other), transactional sex (receiving money/gifts in exchange for sex), forced sex (yes/no), anal sex (yes/no), and frequent travel from home measured as ≥3 days from home per week (yes/no). STIs (chlamydia/gonorrhoea) were tested on endo-cervical swabs using GeneXpert (Cepheid AB, Rontgenvagen 5, Soina Sweden).

### Statistical methods

We analysed data using Stata version 18 (Stata Corp, College Station, TX, USA). Baseline characteristics of the participants were summarised using frequencies and proportions. Chi-squared test for categorical variables and Wilcoxon rank-sum test for non-normally distributed continuous variables were used to assess the differences in baseline demographic, behavioral and sexual risk characteristics between AGYW who were WTP in future clinical trials of LAP and those who were not willing. To estimate baseline factors associated with the WTP in future clinical trials of long-acting PrEP implants, we used a logistic regression model for both unadjusted and adjusted levels; variables for which the association attained **P* *< 0.1 at the unadjusted level were selected for the multivariable model. We also tested for collinearity and for the variables that were found to be highly correlated, i.e., contraceptive use and contraception methods only one of these was selected and included in the final model. The final model was selected based on variables found to be independently associated (P < 0.05) with the outcome. After fitting the final model, adjusted odds ratios (aOR) with p-values and 95% CIs were obtained and reported.

### Ethical considerations

Ethics approval was obtained from the Uganda Virus Research Institute-Research Ethics Committee (GC/127/18/06/658) and the Uganda National Council for Science and Technology/ UNCST (HS 2435). All participants gave written informed consent before participating in the study. The study enrolled emancipated and/or mature minors (14–17 years) who consented for themselves according to national guidelines for the enrolment of emancipated and/or mature minors [25].

## Results

**Fig 1:**
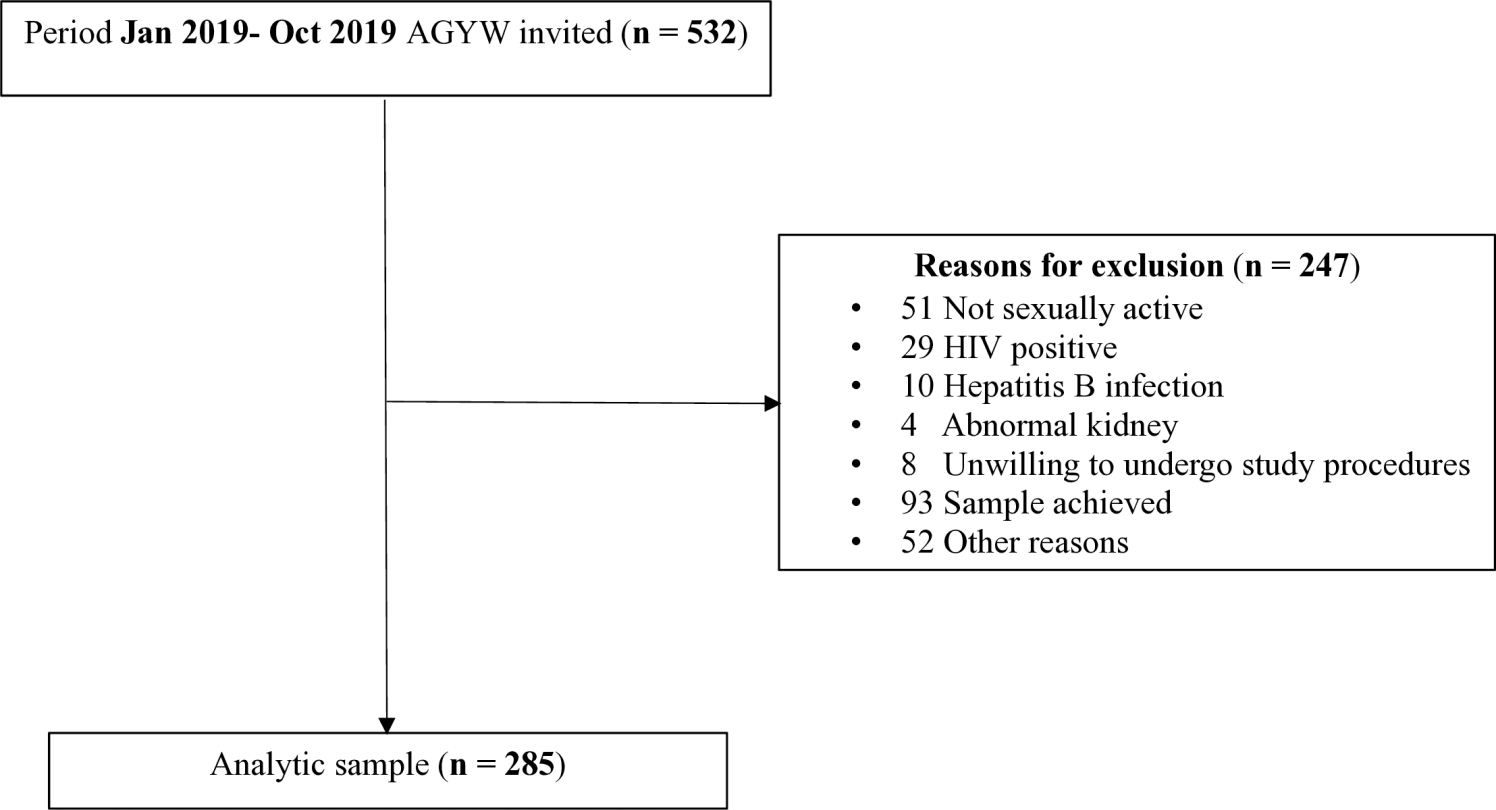
The inclusion and exclusion criteria of adolescent girls and young women (AGYW) for willingness to participate in future clinical trials of long-acting PrEP implant (LAP).

We screened 532 AGYW for study participation, and 247 participants were excluded. A total of 285 AGYW were enrolled in the AGYW cohort, and all were included in this analysis (Fig 1). The median age was 20 years, interquartile range (IQR) 19–22 years and the majority 173 (60.7%) were aged between 20–24 years. Over half of the participants had attained at least secondary level education 156 (54.7%), 163 (57.2%) were single (never married), 201 (70.5%) did not have a steady partner and 103 (36.1%) had no children. A total of 25 (8.8%) were assessed as high-risk alcohol drinkers in the past 12 months, 104 (36.5%) had more than one new male partner, over (209) 73.3% reported consistent condom use, majority 264 (92.6%) engaged in transactional sex and 114 (40.0%) frequently travelled away from home in the past 3 months. The prevalence of STIs (chlamydia and/or gonorrhoea) was 71 (25.3%) (Table 1).

**Table 1:** Demographic characteristics of 285 adolescent girls and young women willing to participate in future clinical trials of long-acting PrEP implant in Uganda (Jan to Oct 2019).

Characteristics	All (n = 285)		Not willing to use LAP implant (n = 154)		Willing to use LAP implant (n = 131)		
	n	(col%)	n	(row%)	n	(row%)	P-Value
**Age (years), median (IQR)**		20 (19-22)		20 (18-21)		20 (19-22)	
**Age group**							
14-19	112	39.3	69	61.6	43	38.4	0.039
20-24	173	60.7	85	49.1	88	50.9	
**Education**							
None/primary	129	45.3	63	48.8	66	51.2	
Secondary/Tertiary	156	54.7	91	58.3	65	41.7	0.109
**Marital status**							
Married	84	29.5	39	46.4	45	53.6	0.241
Separated/divorced	38	13.3	21	55.3	17	44.7	
Single (never married)	163	57.2	94	57.7	69	42.3	
**Partner status**							
With steady partner	84	29.5	39	46.4	45	53.6	
Without a steady partner	201	70.5	115	57.2	86	42.8	0.096
**Employment**							
Sex work	60	21.1	29	48.3	31	51.7	0.761
Hospitality/entertainment	84	29.5	41	54.8	38	45.2	
No Job	68	23.9	37	54.4	31	45.6	
Other	73	25.6	42	57.5	31	42.5	
**Number of children**							
None	103	36.1	67	65.0	36	34.9	0.005
At least one	182	63.9	87	47.8	95	52.2	
**Average income ($)**							
15 or less	216	75.8	108	50.0	108	50.0	0.016
More than 15	69	24.2	46	66.7	23	33.3	
**Alcohol use using AUDIT Tool, past 12 months**							
None	126	44.2	75	59.5	51	40.5	0.159
Low risk drinking	70	24.6	33	47.1	37	52.9	
Moderate/hazardous drinking	64	22.5	30	46.9	34	53.1	
High risk/alcohol dependent	25	8.8	16	64.0	9	36.0	
**Drug use, past 3 month**							
No	241	84.6	132	54.8	109	45.2	0.559
Yes	44	15.4	22	50.0	22	50.0	
**Number of new male partners, past 3 months**							
None/one	181	63.5	108	59.7	73	40.3	
Two or more	104	36.4	46	44.2	58	55.8	0.012
**Condom use, past 3 months**							
Yes	209	73.3	109	52.2	100	47.9	0.290
No	76	26.7	45	59.2	31	40.8	
**Received payment from any male partner, past 3 months**							
No	21	7.4	14	66.7	7	33.3	0.228
Yes	264	92.6	140	53.0	124	46.9	
**Forced sex by any male partner, past 3 months**							0.145
No	218	76.5	123	56.4	95	43.6	
Yes	67	23.5	31	46.3	36	53.7	
**Anal sex with any male, past 3 months**							
No	273	95.8	146	53.5	127	46.5	0.556
Yes	12	4.2	8	66.7	4	33.3	
**Contraceptive use, past 3 months**							
No	127	44.6	80	62.9	47	37.0	0.007
Yes	158	55.4	74	46.8	84	53.2	
**Contraception methods, past 3 months**							
Oral pills	9	3.1	4	44.4	5	55.6	0.001
Injectable	51	17.9	26	50.9	25	49.0	
Implant	47	16.5	12	25.5	35	74.5	
Intra uterine device	2	0.7	1	50.0	1	50.0	
Condoms	41	14.4	27	65.9	14	34.1	
Other	8	2.8	4	50.0	4	50.0	
None	127	44.6	80	62.9	47	37.0	
**Travelled frequently from home, past 3 months**							
No	171	60.0)	99	57.9	72	42.1	0.109
Yes	114	40.0	55	48.3	59	51.8	
**STI diagnosis (chlamydia or gonorrhea)** ^†^							
Negative	210	74.7	107	50.9	103	49.1	0.107
Positive	71	25.3	44	61.9	27	38.0	

† 3 volunteers were not screened for Sexually Transmitted Infection (STIs).

IQR: interquartile range.

AUDIT: Alcohol Use Disorders Identification Test.

**Fig 2:**
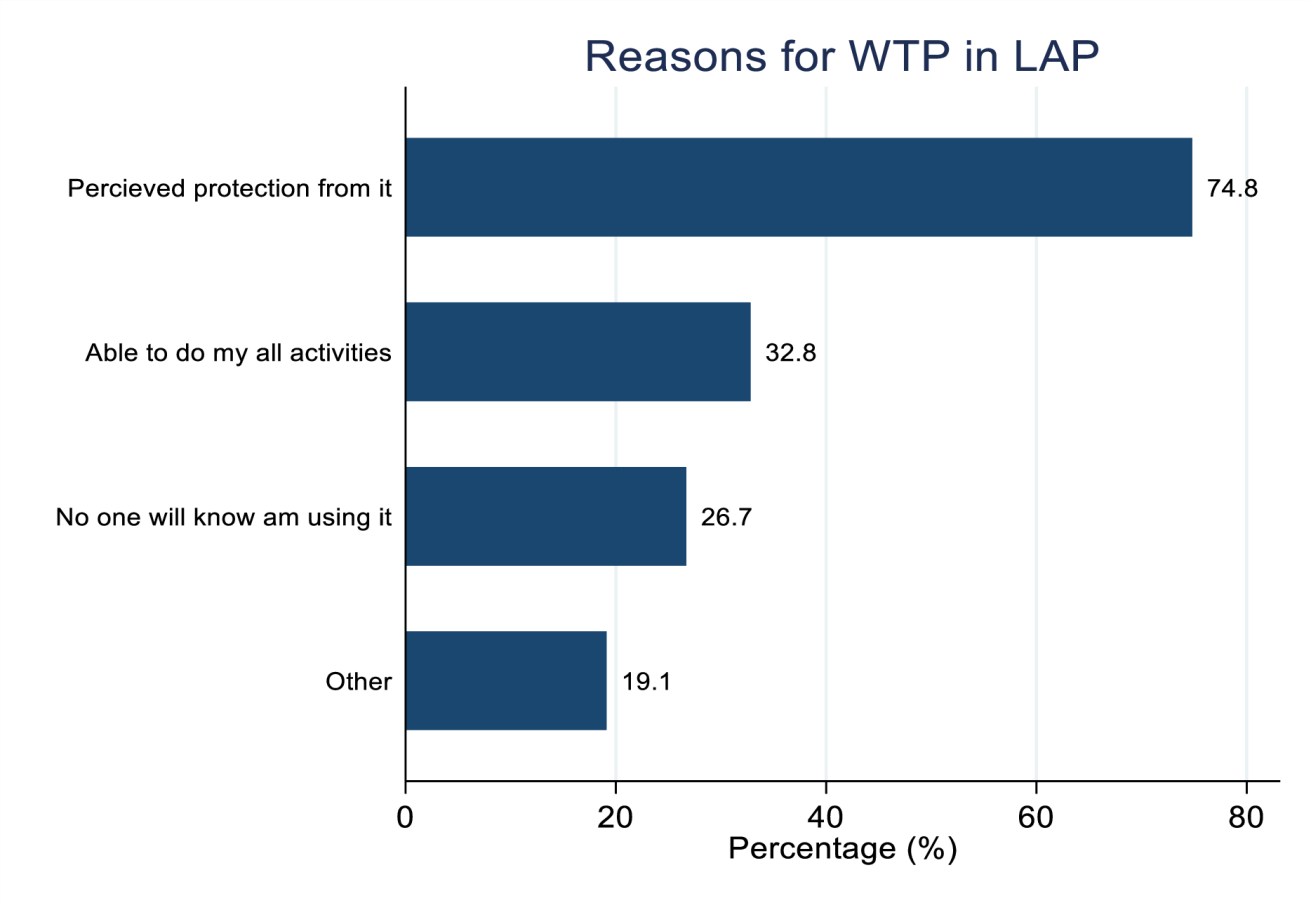
Reasons for the AGYW willing to participate (WTP) in future clinical trials of long-acting PrEP implant.

**Fig 3:**
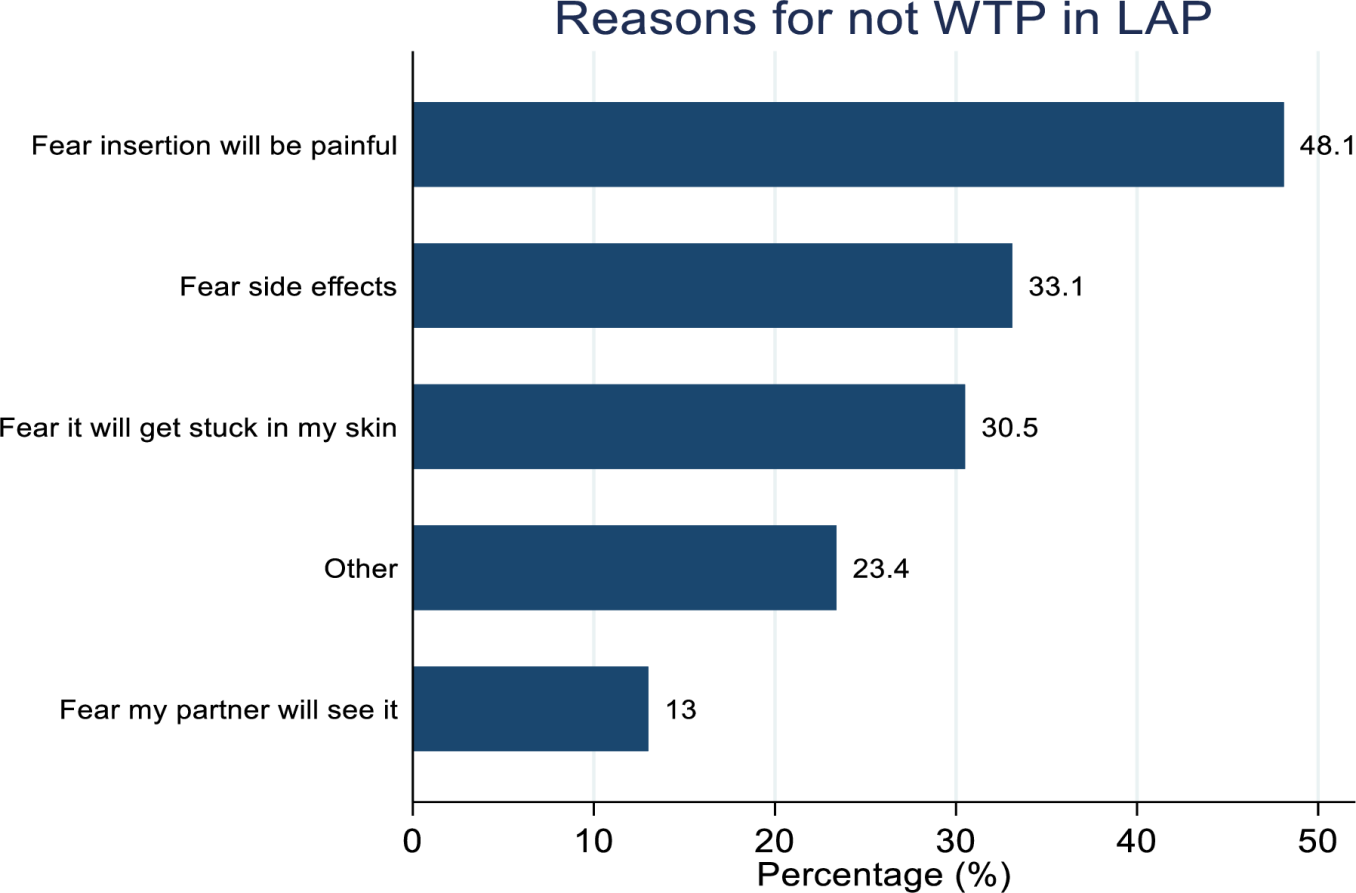
Reasons of AGYW not willing to participate in future clinical trials of long-acting PrEP implant.

Following an education session where participants learned about the attributes of a hypothetical LAP implant and commitments required to participate in a hypothetical clinical trial, the proportion of participants who reported willing to participate (WTP) in an HIV prevention trial of a long-acting PrEP (LAP) implant was 131 (45.9%). WTP in future clinical trials of long-acting PrEP implant was higher among participants aged 20–24 compared to those aged 14–19 (50.9% vs 38.4%; **P* *= 0.039). Participants with at least one child were more likely to report WTP in future clinical trials of long-acting PrEP implant compared to those without children (52.2% vs 34.9%; *P* = 0.005). WTP in future clinical trials of long-acting PrEP implant was higher among participants earning <=$15 compared to those earning > $15 (50.0% vs 33.3%; **P* *= 0.016) while participants who reported having multiple new male partners in the past 3 months were more WTP in future clinical trials of long-acting PrEP implant when compared to those with none/one (55.8% vs 40.3%; *P* = 0.012). Additionally, participants who were using contraceptives were more WTP in future clinical trials of long-acting PrEP implant compared to those not using (53.2% vs 37.0%; **P* *= 0.007) (Table 1). The main reasons for willingness to participate in clinical trials of LAP implant among the 131 who expressed WTP included perceived protection from the implant 98 (74.8%) and the ability to carry out activities without worrying about the LAP implant 43 (32.8%) (Fig 2), while fear of perceived painful insertion 74 (48.1%) and fear of side effects of the implant 51 (33.1%) were the main reasons reported among 154 (54.0%) participants who were not willing to participate in clinical trials of LAP (Fig 3).

Regarding clinical trial attributes, overall willingness to participate in HIV prevention trials was high (98.0%). However, willingness to participate in trials of a long-acting PrEP implant was low 131 (45.9%) compared to trials of other HIV prevention methods like vaccine(s) 233 (81.8%) and injectable PrEP 199 (69.8%).

In the adjusted logistic regression analysis, WTP was associated with earning an average weekly income > $15 (aOR 0.46, 95%CI 0.25-0.84, *P* = 0.013), having 2 or more new male partners in the past 3 months (aOR 1.84, 95% CI 1.09-3.11, *P* = 0.022) and using contraceptives (aOR 1.69, 95%CI 1.00-2.85, *P* = 0.047) after adjusting for age, education level, marital status, number of children and STI diagnosis (Table 2).

**Table 2:** Factors associated with willingness to participate in future clinical trials of long-acting PrEP implant among adolescent girls and young women in Uganda (Jan to Oct 2019), N = 285.

Characteristic	Un Adjusted Odds Ratio (uOR)		Adjusted Odds Ratio (aOR)	
	OR (95% CI)	P-Value	OR (95% CI)	P-Value
**Age group**				
14-19	Ref		Ref	
20-24	1.66 (1.02-2.69)	0.040	1.27 (0.72-2.25)	0.396
**Education**				
None/Primary	Ref			
Secondary/Tertiary	0.68 (0.42-1.09)	0.110		
**Partner status**				
With steady partner	Ref			
Without a steady partner	0.64 (0.38-1.08)	0.097		
**Number of children**				
None	Ref		Ref	
At least one	2.03 (1.23-3.34)	0.005	1.55 (0.86-2.78)	0.138
**Average income ($)**				
15 or less	Ref		Ref	
More than 15	0.50 (0.28-0.88)	0.017	0.46 (0.25-0.84)	0.013
**Alcohol use using AUDIT Tool**, **past 12 months**				
None	Ref			
Low risk drinking	1.64 (0.91-2.97)	0.096		
Moderate/hazardous drinking	1.66 (0.90-3.05)	0.099		
High risk/alcohol dependent	0.82 (0.33-2.01)	0.676		
**Drug use, past one month**				
No	Ref			
Yes	1.21 (0.63-2.30)	0.560		
**Number of new male partners, past 3 months**				
None/one	Ref		Ref	
Two or more	1.86 (1.14-3.03)	0.012	1.84 (1.09-3.11)	0.022
**Contraceptive use, past 3 months**				
No	Ref			
Yes	1.93 (1.19-3.11)	0.007	1.69 (1.00-2.85)	0.047
**Contraception methods, past 3 months**				
None	Ref			
Oral pills	2.12 (0.54-8.31)	0.278		
Injectable	1.63 (0.84-3.15)	0.141		
Implant	4.96 (2.34-10.48)	0.000		
Intra uterine device	1.70 (0.10-27.85)	0.709		
Condoms	0.88 (0.42-1.84)	0.741		
Other	1.70 (0.40-7.12)	0.467		
**STI diagnosis (chlamydia or gonorrhea)**				
Negative	Ref		Ref	
Positive	0.63 (0.37-1.10)	0.109	0.63 (0.35-1.12)	0.119
**Received payment from any male partner, past 3 months**				
No	Ref			
Yes	1.77 (0.69-4.52)	0.233		
**Forced sex by any male partner, past 3 months**				
No	Ref			
Yes	1.50 (0.86-2.60)	0.146		
**Travelled frequently from home, past 3 months**				
No	Ref			
Yes	1.47 (0.91-2.37)	0.110		

CI: confidence interval, Ref: reference.

STI; Sexually transmitted infections.

## Discussion

Our findings showed that almost half of enrolled Ugandan AGYW at risk of HIV acquisition are willing to participate in future trials of a long-acting PrEP implant. However, WTP in future trials of the PrEP implant has not been extensively documented, despite pre-clinical development of subdermal biodegradable tenofovir alafenamide and cabotegravir reservoir implants [19,20]. This level of interest suggests a readiness among AGYW to consider biomedical innovations that could offer more discreet and longer-term protection against HIV. In contrast, WTP in HIV vaccine trials has been widely studied among key populations (e.g., female sex workers, men who have sex with men, and fisherfolk) in East Africa with studies reporting high WTP (81% - 89%) [26–29]. However, it is important to note that the high reported WTP does not always translate into high actual participation [30]. This gap highlights the need for trial designs that not only assess initial willingness but additionally address potential barriers to retention and adherence throughout the study process.

The percentage of WTP that we report could be attributed to the high HIV risk perception among AGYW who reported multiple new male partners and consequently a strong desire for protection against HIV. Studies conducted elsewhere have demonstrated a positive association between increased HIV risk perception and higher WTP in HIV prevention clinical trials as well as a greater adoption of preventive measures [31–33]. In line with this, the most commonly cited reason for WTP among AGYW in our study was hope of protection against HIV, which is consistent with findings from a study assessing WTP for HIV vaccine trials among FSWs in Kampala Uganda [28]. These findings emphasize the need to design communication and strategies that align with individual risk perception especially targeting those who may not perceive themselves to be at risk despite being in a high-risk situation.

The lower WTP for PrEP implant compared to WTP observed in HIV vaccine studies may be linked to the implant not being a common mode of drug delivery in health care settings, apart from reproductive health clinics. In our cohort, about half of the participants had limited experience with available contraceptive methods, such as implants, which may also pose a barrier to their willingness to adopt implantable PrEP technology. This aligns with existing literature indicating that lack of awareness and familiarity with new interventions, such as contraceptive implants, can lead to lower acceptance rates [34]. Additionally, studies have also shown that patients’ understanding of new technologies and clinical trial procedures significantly impacts their willingness to participate [35].

We previously demonstrated in a study that assessed preference for five novel biomedical HIV prevention methods (oral PrEP, injectable PrEP, vaginal ring, PrEP implant, preventative HIV vaccine) in the same cohort of AGYW that 14% indicated a PrEP implant as their most preferred HIV prevention method [36]. This low proportion was among those who were already familiar with using the contraceptive implant which aligns with findings from other studies conducted among AGYW [10,11]. AGYW reported fear of painful insertion and side effects as barriers to WTP. To address these concerns, study information and education about future LAP implants should include messages on how undesirable product attributes are handled including actual provision of counselling and health care support for potential side effects. Providing this information may enhance participation in future LAP implant trials.

AGYWs who reported multiple new male partners in the past 3 months were more willing to participate in future PrEP implant trials highlighting the potential of long-acting prevention tools to meet the needs of those at greater risk of HIV. This suggests that risk perception can be a powerful motivator for adopting biomedical prevention strategies that offer longer durations of protection from HIV. These finding align with studies conducted among key populations in Uganda and Kenya, which demonstrated high WTP in HIV vaccine trials of products that offered long durations of protection from HIV [27–29]. Considering AGYW with multiple sexual partners, PrEP implants could offer a discreet option which is a desirable product attribute.

In addition, findings from other HIV prevention studies among AGYW at risk of HIV infection in Sub Saharan Africa indicate challenges with oral PrEP uptake, compliance [37,38] and consistent condom use [38,39]; under such circumstances, a long acting and discreet product like a PrEP implant which would not require frequent insertion or clinic visit, would be ideal. Other studies have also shown that due to socially constructed gender inequalities, many AGYW may not be able to negotiate for safe sex due to low decision-making power during intimate relationships as most decisions about sex are made by men [40–42]. A long-acting and discreet product like a PrEP implant which offers long-term protection against HIV is a solution in situations where condom negotiation is not possible. It also has the added advantage of less anxiety over HIV acquisition leading to improved mental health.

Our finding that WTP in future PrEP implant trials was higher among AGYW using contraception is consistent with prior studies conducted in other settings [36,43–45]. Implants have been widely used to successfully deliver contraception, and in our study, implantable and injectable contraception were the most commonly used methods among AGYW. This suggests that AGYW may be more open to adopt similar biomedical products for HIV prevention. Familiarity with the contraceptive implants may have contributed to greater WTP, as prior use of these technologies likely reduces uncertainty or fear related to similar interventions. As awareness and uptake of biomedical prevention tools increase, willingness to participate in future trials of related products may also rise.

Our findings among those using contraception indicate that clinical trial requirements to use an effective contraceptive during the trial may be met with ease. However, actual participation in future trials of PrEP implants may pose challenges for those already using contraceptive implants as it may not be desirable for some potential trial participants to have two separate implants, one for contraception and the other for HIV PrEP. This highlights the need for the development of multi-purpose prevention products that provide simultaneous protection against both HIV and unintended pregnancy, which could further enhance product acceptability and uptake.

We found that participants with higher income levels were less likely to show WTP in future trials of PrEP implants. This suggests that socio-economic status may influence how AGYW assess the need or perceived value of participating in biomedical HIV prevention research. AGYW with higher income may perceive themselves as having greater access to existing HIV prevention methods whether through private clinics, self-funded transportation or greater autonomy in healthcare decisions and may not feel compelled to participate in clinical trials. Also, higher income individuals may perceive less personal benefit from clinical trial participation or express greater concerns about potential risks. This finding adds depth to existing literature, which has reported mixed associations between income and PrEP use across different settings. Findings from a study conducted among high-risk adolescent boys and young men in Uganda reported that participants that earned more than 27 dollars per week were less willing to use PrEP compared to those who earned less than 27 dollars [46]. Relatedly, a study conducted among men who have sex with men in the US also revealed similar results indicating that those who earned lower income were more likely to use PrEP compared to those who earned higher income [47], possibly reflecting a strong reliance on free or research supported prevention options. These findings however, contradict studies conducted among high risk MSM in China and Boston which showed that those with higher incomes were more likely to use PrEP in the future compared to those with lower income levels [48,49]. These varying results highlight how economic status can affect not only access to HIV prevention but also how individuals prioritize and perceive their need for such interventions. While these studies provide insight into the relationship between income and PrEP use, there is limited literature specifically examining the association between income and WTP in clinical trials for long -acting PrEP. Hence more research is needed to explore how socioeconomic factors influence participation in PrEP trials and the adoption of new HIV prevention technologies.

### Strengths and limitations

Firstly, our study was limited by the use of non-probability sampling methods that are prone to selection bias which affects the generalizability of findings. Secondly, a PrEP implant is still in the development stage, the information provided to participants in the hypothetical trial about the product’s attributes would likely differ from the descriptions used in the actual future trials. Thirdly, there is a potential for social desirability bias, particularly in responses to sensitive questions related to sexual behaviour, substance use, and willingness to participate. Despite efforts to ensure privacy and confidentiality during data collection, participants may have underreported stigmatized behaviours or overreported socially desirable ones, which could have impacted the accuracy of self-reported data. Despite these limitations, our study was (to the best of our knowledge) the first in Uganda to show the level of willingness of AGYW at high risk of HIV infection to participate in future PrEP implant HIV prevention trials. It adds to the knowledge on AGYW and HIV prevention strategies, especially in the era of long-acting PrEP. Additionally, with the growing concept of multi-purpose prevention products, our study contributes to the expanding body of literature on people’s attitudes and possible willingness to use multi-purpose PrEP implants that prevent both HIV and pregnancy.

## Conclusions

Nearly half of AGYW expressed WTP in future trials of the PrEP implant. While this indicates a promising level of initial interest in novel HIV prevention methods, it also highlights the need for targeted interventions to address barriers to acceptability and enhance uptake. Experience using contraceptive methods with a similar mode of delivery or that offer long duration of protection may increase WTP. Further research is needed to explore other factors that may impact WTP, as understanding AGYW’s perspectives will be crucial for the successful implementation of future trials.

## Supporting information

S1 Data Supplementary file dataset. (XLSX)

## References

[1] UNAIDS. UNAIDS 2024 epidemiological estimates. 2024.

[2] UNAIDS. HIV and adolescent girls and young women—2024 Global AIDS Update. 2024. Available from: https://www.unaids.org/sites/default/files/media_asset/2024-unaids-global-aids-update-adolescent-girls-young-women_en.pdf

[3] CDC. Global HIV and TB. 2024. Available from: https://www.cdc.gov/global-hiv-tb/php/success-stories/dreams-saving-lives.html

[4] Dadzie LK, Agbaglo E, Okyere J, Aboagye RG, Arthur-Holmes F, Seidu A-A, et al. Self-reported sexually transmitted infections among adolescent girls and young women in sub-Saharan Africa. Int Health. 2022;14(6):545–53. doi: 10.1093/inthealth/ihab088 35134172 PMC9623488

[5] Kawuma R, Lunkuse JF, Ssembajjwe W, Kayesu I, Price MA, Brickley DB, et al. “I fear those things”: non-uptake of contraceptives, and barriers to use among adolescent girls and young women at high risk of HIV infection in Kampala, Uganda. Front Reprod Health. 2023;5:1198672. doi: 10.3389/frph.2023.1198672 37649966 PMC10465063

[6] Singer SE, Wechsberg WM, Kline T, Browne FA, Howard BN, Carney T, et al. Binge drinking and condom negotiation behaviours among adolescent girls and young women living in Cape Town, South Africa: sexual control and perceived personal power. BMC Public Health. 2023;23(1):2282. doi: 10.1186/s12889-023-17188-0 37980472 PMC10657119

[7] Mugwanya KK, Pintye J, Kinuthia J, Abuna F, Lagat H, Begnel ER, et al. Integrating preexposure prophylaxis delivery in routine family planning clinics: A feasibility programmatic evaluation in Kenya. PLoS ONE. 2019;16(9):e1002885. 10.1371/journal.pmed.1002885 PMC6719826 31479452

[8] Mayanja Y, Kamacooko O, Lunkuse JF, Muturi-Kioi V, Buzibye A, Omali D, et al. Oral pre-exposure prophylaxis preference, uptake, adherence and continuation among adolescent girls and young women in Kampala, Uganda: a prospective cohort study. J Int AIDS Soc. 2022;25(5):e25909. doi: 10.1002/jia2.25909 35543110 PMC9092160

[9] Tapsoba J de D, Cover J, Obong’o C, Brady M, Cressey TR, Mori K, et al. Continued attendance in a PrEP program despite low adherence and non-protective drug levels among adolescent girls and young women in Kenya: Results from a prospective cohort study. PLoS Med. 2022;19(9):e1004097. doi: 10.1371/journal.pmed.1004097 36095005 PMC9521917

[10] Kayesu I, Mayanja Y, Nakirijja C, Machira YW, Price M, Seeley J, et al. Uptake of and adherence to oral pre-exposure prophylaxis among adolescent girls and young women at high risk of HIV-infection in Kampala, Uganda: A qualitative study of experiences, facilitators and barriers. BMC Womens Health. 2022;22(1):440. doi: 10.1186/s12905-022-02018-z 36357920 PMC9648457

[11] Muhumuza R, Ssemata AS, Kakande A, Ahmed N, Atujuna M, Nomvuyo M, et al. Exploring Perceived Barriers and Facilitators of PrEP Uptake among Young People in Uganda, Zimbabwe, and South Africa. Arch Sex Behav. 2021;50(4):1729–42. doi: 10.1007/s10508-020-01880-y 33954824 PMC8213546

[12] Jani N, Mathur S, Kahabuka C, Makyao N, Pilgrim N. Relationship dynamics and anticipated stigma: Key considerations for PrEP use among Tanzanian adolescent girls and young women and male partners. PLoS One. 2021;16(2):e0246717. doi: 10.1371/journal.pone.0246717 33596216 PMC7888654

[13] Bergam S, Harrison AD, Benghu N, Khumalo S, Tesfay N, Exner T, et al. Women’s Perceptions of HIV- and Sexuality-Related Stigma in Relation to PrEP: Qualitative Findings from the Masibambane Study, Durban, South Africa. AIDS Behav. 2022;26(9):2881–90. doi: 10.1007/s10461-022-03632-6 35218452 PMC9378426

[14] WHO. WHO recommends long-acting cabotegravir for HIV prevention. 2021. Available from: https://www.who.int/news/item/28-07-2022-who-recommends-long-acting-cabotegravir-for-hiv-prevention

[15] WHO. WHO recommends the dapivirine vaginal ring as a new choice for HIV prevention for women at substantial risk of HIV infection. 2021. Available from: https://www.who.int/news/item/26-01-2021-who-recommends-the-dapivirine-vaginal-ring-as-a-new-choice-for-hiv-prevention-for-women-at-substantial-risk-of-hiv-infection

[16] MOH. Consolidated guidelines for the prevention and treatment of HIV and AIDS in Uganda. 2022.

[17] Landovitz RJ, Li S, Eron JJ Jr, Grinsztejn B, Dawood H, Liu AY, et al. Tail-phase safety, tolerability, and pharmacokinetics of long-acting injectable cabotegravir in HIV-uninfected adults: a secondary analysis of the HPTN 077 trial. Lancet HIV. 2020;7(7):e472–81. doi: 10.1016/S2352-3018(20)30106-5 32497491 PMC7859863

[18] Gilead’s Twice-Yearly Lenacapavir Demonstrated 100% Efficacy and Superiority to Daily Truvada for HIV Prevention [press release]. 2024.

[19] Massud I, Krovi A, Nishiura K, Ruone S, Li L, Holder A, et al. Safety and efficacy of a biodegradable implant releasing tenofovir alafenamide for vaginal protection in a macaque model. J Antimicrob Chemother. 2022;77(11):2964–71. doi: 10.1093/jac/dkac252 35913838 PMC10205616

[20] Karunakaran D, Simpson SM, Su JT, Bryndza-Tfaily E, Hope TJ, Veazey R, et al. Design and Testing of a Cabotegravir Implant for HIV Prevention. J Control Release. 2021;330:658–68. doi: 10.1016/j.jconrel.2020.12.024 33347943 PMC7906957

[21] Little KM, Flomen L, Hanif H, Anderson SM, Thurman AR, Clark MR, et al. HIV Pre-exposure Prophylaxis Implant Stated Preferences and Priorities: Results of a Discrete Choice Experiment Among Women and Adolescent Girls in Gauteng Province, South Africa. AIDS Behav. 2022;26(9):3099–109. doi: 10.1007/s10461-022-03658-w 35360893 PMC9371991

[22] Vandepitte J, Bukenya J, Weiss HA, Nakubulwa S, Francis SC, Hughes P, et al. HIV and other sexually transmitted infections in a cohort of women involved in high-risk sexual behavior in Kampala, Uganda. Sex Transm Dis. 2011;38(4):316–23. doi: 10.1097/olq.0b013e3182099545 23330152 PMC3920055

[23] Barrett SE, Teller RS, Forster SP, Li L, Mackey MA, Skomski D, et al. Extended-Duration MK-8591-Eluting Implant as a Candidate for HIV Treatment and Prevention. Antimicrob Agents Chemother. 2018;62(10):e01058-18. doi: 10.1128/AAC.01058-18 30012772 PMC6153840

[24] WHO. AUDIT: the Alcohol Use Disorders Identification Test: guidelines for use in primary health care/ Thomas F. Babor … [et al.]. 2nd ed. Geneva: World Health Organization; 2001.

[25] UNCST. National guidelines for research involving humans as research participants. 2014. Available from: https://iuea.ac.ug/sitepad-data/uploads//2021/03/Human-Subjects-Protection-Guidelines-July-2014.pdf

[26] Nanvubya A, Mpendo J, Ssetaala A, Kidega W, Sigirenda S, Nielsen L, et al. Willingness to participate in HIV vaccine efficacy trials in a population of fishing communities, Uganda. (1742-4690 (Electronic)).

[27] Mutisya EM, Mutua G, Nyasani D, Nduta H, Kabuti RW, Muturi-Kioi V. Willingness to participate in future HIV vaccine trials among men who have sex with men and female sex workers living in Nairobi, Kenya. BMC Infect Dis. 2020;15(8):e0238028. 10.1371/journal.pone.0238028 PMC7444816 32834018

[28] Mayanja Y, Abaasa A, Namale G, Price MA, Kamali A. Willingness of female sex workers in Kampala, Uganda to participate in future HIV vaccine trials: a case control study. BMC Public Health. 2020;20(1):1789. doi: 10.1186/s12889-020-09932-7 33239018 PMC7686944

[29] Asiki G, Abaasa A, Ruzagira E, Kibengo F, Bahemuka U, Mulondo J, et al. Willingness to participate in HIV vaccine efficacy trials among high risk men and women from fishing communities along Lake Victoria in Uganda. Vaccine. 2013;31(44):5055–61. doi: 10.1016/j.vaccine.2013.08.080 24021306 PMC4506465

[30] Nyasani DK, Mutua GN, Sajabi RM, Ng’ang’a JW, Gachie JN, Maina AM, et al. Reported willingness to participate in a hypothetical HIV vaccine trial and its translation to actual participation among healthy adults-Experience from Kenya. PLoS One. 2018;13(11):e0206656. doi: 10.1371/journal.pone.0206656 30388145 PMC6214541

[31] Sebatta DE, Siu G, Nabeta HW, Anguzu G, Walimbwa S, Lamorde M, et al. “You would not be in a hurry to go back home”: patients’ willingness to participate in HIV/AIDS clinical trials at a clinical and research facility in Kampala, Uganda. BMC Med Ethics. 2020;21(1):77. doi: 10.1186/s12910-020-00516-z 32831090 PMC7446203

[32] Sewell WC, Patel RR, Blankenship S, Marcus JL, Krakower DS, Chan PA, et al. Associations Among HIV Risk Perception, Sexual Health Efficacy, and Intent to Use PrEP Among Women: An Application of the Risk Perception Attitude Framework. AIDS Educ Prev. 2020;32(5):392–402. doi: 10.1521/aeap.2020.32.5.392 33112674 PMC8049455

[33] Yellin H, Levy ME, Magnus M, Kuo I, Siegel M. HIV Risk Perception, Willingness to Use PrEP, and PrEP Uptake Among Young Men who have Sex with Men in Washington, DC. AIDS Behav. 2023;27(9):2844–54. doi: 10.1007/s10461-023-04008-0 36807246 PMC10439971

[34] Kff. Contraceptive Implants. 2019. Available from: https://www.kff.org/womens-health-policy/fact-sheet/contraceptive-implants/

[35] von Itzstein MS, Railey E, Smith ML, White CB, Sledge GW Jr, Howell JR, et al. Patient familiarity with, understanding of, and preferences for clinical trial endpoints and terminology. Cancer. 2020;126(8):1605–13. doi: 10.1002/cncr.32730 31967687 PMC7276207

[36] Mayanja Y, Kayesu I, Kamacooko O, Lunkuse JF, Muturi-Kioi V, Price M, et al. Preference for novel biomedical HIV pre-exposure prophylaxis methods among adolescent girls and young women in Kampala, Uganda: a mixed methods study. Front Public Health. 2024;12:1369256. doi: 10.3389/fpubh.2024.1369256 38846614 PMC11153736

[37] Velloza J, Donnell D, Hosek S, Anderson PL, Chirenje ZM, Mgodi N, et al. Alignment of PrEP adherence with periods of HIV risk among adolescent girls and young women in South Africa and Zimbabwe: a secondary analysis of the HPTN 082 randomised controlled trial. Lancet HIV. 2022;9(10):e680–9. doi: 10.1016/S2352-3018(22)00195-3 36087612 PMC9530001

[38] Mayanja Y, Kamacooko O, Lunkuse JF, Muturi-Kioi V, Buzibye A, Omali D, et al. Oral pre-exposure prophylaxis preference, uptake, adherence and continuation among adolescent girls and young women in Kampala, Uganda: a prospective cohort study. J Int AIDS Soc. 2022;25(5):e25909. doi: 10.1002/jia2.25909 35543110 PMC9092160

[39] Calabrese SK, Dovidio JF, Tekeste M, Taggart T, Galvao RW, Safon CB, et al. HIV Pre-Exposure Prophylaxis Stigma as a Multidimensional Barrier to Uptake Among Women Who Attend Planned Parenthood. J Acquir Immune Defic Syndr. 2018;79(1):46–53. doi: 10.1097/QAI.0000000000001762 29847480 PMC6092222

[40] Izudi J, Okello G. Low condom use at the last sexual intercourse among university students in sub-Saharan Africa: evidence from a systematic review and meta-analysis. BMC Public Health. 2022;17(8):e0272692. 10.1371/journal.pone.0272692 PMC9365151 35947583

[41] Duffy L. Culture and context of HIV prevention in rural Zimbabwe: the influence of gender inequality. J Transcult Nurs. 2005;16(1):23–31. doi: 10.1177/1043659604270962 15608096

[42] Small E, Nikolova SP, Zhou Y, Okumu M. Exploring factors associated with HIV secondary stigma among adolescents and young adults in Uganda: A cross-sectional study. Glob Public Health. 2022;17(4):526–37. doi: 10.1080/17441692.2020.1869286 33406003 PMC12184802

[43] Calabrese SK, Galvao RW, Dovidio JF, Willie TC, Safon CB, Kaplan C, et al. Contraception as a Potential Gateway to Pre-Exposure Prophylaxis: US Women’s Pre-Exposure Prophylaxis Modality Preferences Align with Their Birth Control Practices. AIDS Patient Care STDS. 2020;34(3):132–46. doi: 10.1089/apc.2019.0242 32202930 PMC7087409

[44] Ngure K, Velloza J, Patel RC, Mugo NR, Bukusi EA, Haberer JE, et al. Alignment of PrEP use and effective contraceptive use among East African women in HIV serodiscordant partnerships. Int J STD AIDS. 2020;31(13):1263–71. doi: 10.1177/0956462420951501 32998640 PMC7982141

[45] Little KM, Hanif H, Anderson SM, Clark MR, Gustafson K, Doncel GF. Preferences for Long-Acting PrEP Products Among Women and Girls: A Quantitative Survey and Discrete Choice Experiment in Eswatini, Kenya, and South Africa. AIDS Behav. 2024;28(3):936–50. doi: 10.1007/s10461-023-04202-0 37971614 PMC10896879

[46] Agwang W, Nangendo J, Nabikande S, Okello T, Tusabe J, Semitala FC, et al. Factors associated with willingness to take Pre-Exposure Prophylaxis (PrEP) among high-risk adolescent boys and young men in Masese fishing community, Uganda. PLOS Glob Public Health. 2023;3(6):e0001191. doi: 10.1371/journal.pgph.0001191 37289700 PMC10249897

[47] Kamitani E, Wichser ME, Mizuno Y, DeLuca JB, Higa DH. What Factors Are Associated With Willingness to Use HIV Pre-exposure Prophylaxis (PrEP) Among U.S. Men Who Have Sex With Men Not on PrEP? A Systematic Review and Meta-analysis. J Assoc Nurses AIDS Care. 2023;34(2):135–45. doi: 10.1097/JNC.0000000000000384 36563302 PMC10184317

[48] Zhang Y, Peng B, She Y, Liang H, Peng H-B, Qian H-Z, et al. Attitudes toward HIV pre-exposure prophylaxis among men who have sex with men in western China. AIDS Patient Care STDS. 2013;27(3):137–41. doi: 10.1089/apc.2012.0412 23425017 PMC3595955

[49] Mimiaga MJ, Case P, Johnson CV, Safren SA, Mayer KH. Preexposure antiretroviral prophylaxis attitudes in high-risk Boston area men who report having sex with men: limited knowledge and experience but potential for increased utilization after education. J Acquir Immune Defic Syndr. 2009;50(1):77–83. doi: 10.1097/QAI.0b013e31818d5a27 19295337 PMC2659469

